# Physical Principles of a Piezo Accelerometer Sensitive to a Nearly Constant Signal

**DOI:** 10.3390/s18103258

**Published:** 2018-09-28

**Authors:** Valery Gupalov, Alexander Kukaev, Sergey Shevchenko, Egor Shalymov, Vladimir Venediktov

**Affiliations:** 1 Laser Measurement and Navigation Systems Department, Electrotechnical University “LETI”, St. Petersburg 197376, Russia; vigupalov@yandex.ru (V.G.); askukaev@gmail.com (A.K.); shev1989@yandex.ru (E.S.); syshevchenko@mail.ru (S.S.); 2 Quantum Electronics Department, Faculty of Physics, Saint Petersburg State University, St. Petersburg 198504, Russia

**Keywords:** accelerometer, apparent acceleration, navigation systems

## Abstract

The paper considers the construction of a piezoelectric accelerometer capable of measuring constant linear acceleration. A number of designs are proposed that make it possible to achieve high sensitivity with small dimensions and a wide frequency band (from 10^−5^ Hz). The finite element model of the proposed design was investigated, and its output characteristic and scale factor (36 mV/g) were obtained.

## 1. Introduction

Every year, the requirements for various inertial sensors and, in particular, for accelerometers, are becoming increasingly strict and complex. Previously, the key indicators were accuracy and sensitivity, but today an increasingly important role is assigned to dimensions, cost, impact resistance, and a number of other parameters. As a result, new design concepts of accelerometers were developed, such as MEMS accelerometers, for example. It should be noted that today, with a growing interest in unmanned aerial vehicles and cars, as well as smart devices and instruments, the sensor market is experiencing significant growth, which opens up new market niches. Therefore, the consumer is offered a wide range of different sensors that meet some or other requirements. However, there are applications that require the simultaneous meeting of a number of requirements. For example, for creating autonomous navigation systems an important feature is the ability to measure apparent acceleration. Popular MEMS accelerometers meet this requirement, but for them the lowest detection limit is of the order of 10^−4^ g [1], which is not enough for navigational systems of tactical accuracy class. On the other hand, there are piezoelectric accelerometers that have a low detection limit (10^−5^–10^−6^ g) and shock resistance, but are limited from below by a frequency range of the order of 0.1 Hz [2]. In this paper, we propose a new design concept of the piezoelectric accelerometer to combine the advantages of the two abovementioned types of transducers.

## 2. Principle of Operation

**Figure 1:**
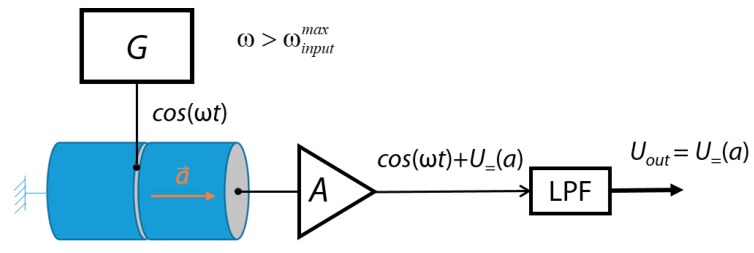
Design concept of a proposed microaccelerometer. G: generator; A: amplifier; LPF: low-pass filter.

The main reason limiting the bandwidth of piezoelectric accelerometers is the charge leakage effect. Therefore, if the piezoelectric effect is artificially transferred from the area of constant signals to the variable domain, it is possible to extend the lower limit of the accelerometer sensitivity band. Generally, authors do not see any limitations to measure even constant signals, but in the case of on-Earth applications, where *g* values change during the day due to the movement of astronomical bodies, let us take a lower detecting frequency limit of 1/86,400 s or approximately 10^−5^ Hz. For such measurements, it is proposed to use the construction shown in Figure 1 [3].

Here, the exciting generator *G* creates a harmonic signal with frequency ω and directs it to the piezoelectric transducer, divided into two parts: exciting and sensitive. Each of them is a cylinder made of piezo ceramics, with longitudinal polarization. One of the sides of the converter exciting part is rigidly fixed, and the second is rigidly connected to the sensitive part through the electrode to which the generator is connected. The second part also acts as a test mass (if necessary, to increase the sensitivity of the sensor, the free end of this converter can be additionally loaded). Under the action of the exciting signal—in which the frequency ω should be higher than the maximum frequency of the acceleration to be measured—the converter performs harmonic oscillations and a corresponding signal appears at the output of its sensitive part. The latter goes to the amplifier *A* and then to a low-pass filter (LPF), which forms a constant voltage *U_out_* equal to the midline of the harmonic signal. It should be noted that for an excitation signal any alternating signal, including centered random noise, might be used.

Under the action of acceleration, a rod—formed by piezoelectric transducers—deforms and an additional charge proportional to the actual acceleration is excited on the electrodes of the sensitive part. As a result, the midline of the harmonic output signal will change its position and, after that, the value of the voltage *U_out_* taken from the filter output will change. In this case, the acceleration measurement range will be primarily determined by the parameters of the amplifier and can be easily adapted to the requirements of a particular consumer or even changed during the sensor operation. The output signal is presented by a constant voltage, convenient for further processing. It is necessary to emphasize that the piezoelectric part is always in a dynamic mode. Literally, it means that the effect of a charge leakage is almost neglected, because its time constant is much higher than the one of the excitation signal. By the time that the charge leaks by a significant value, the total output voltage already changes its sign. 

**Figure 2:**
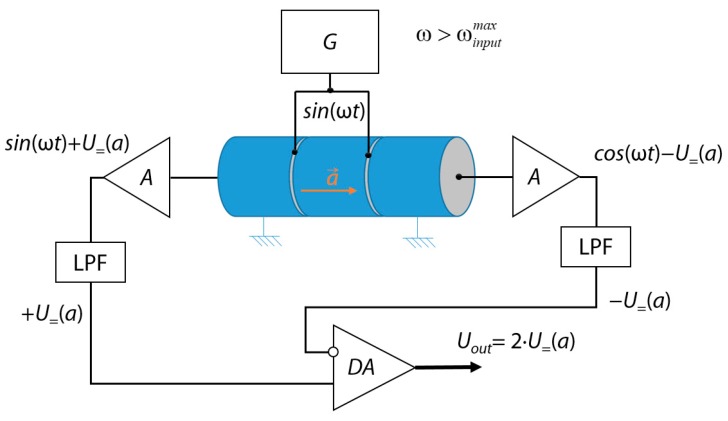
Differential piezoelectric accelerometer design concept. DA: differential amplifier.

If necessary, this scheme can be modified to organize a differential operation mode. Such an option is shown in Figure 2 [4].

In this case, the piezoelectric transducer consists of three parts: one exciter (in the center) and two rigidly fixed receivers (at the edges). Each of their outputs is also connected to a charge amplifier and a low-pass filter connected in series. The outputs of the latter are connected to the inputs of the differential amplifier (*DA*), which forms the differential output signal. Such a design provides high temperature stability of the sensor.

Let us now consider the influence of the piezoelectric transducer dimensions on the sensor characteristics. An informative signal is the voltage *U_out_*, which arises from the deformation of the converter under the action of acceleration. We can write the following chain of expressions [5]:

(1) Uout=QC=d33F(εε0Sh)=d33εε0FSh=d33εε0maSh=d33εε0ρhSaSh=Kph2a 

where *Q* is the charge generated by the acceleration, *C* is the capacitance of the piezoelectric transducer, *d*_33_ is the piezoelectric module, *F* is the force acting on the piezoelectric transducer, ε, ρ are the permittivity and density of the piezoelectric transducer material, respectively, *S*, *h*, *m* is its cross section, thickness, and mass, respectively, ε_0_ = 8.85 × 10^–12^ F/m is the dielectric constant of vacuum, *Kp* = *d*_33_ρ/εε_0_ is the coefficient of proportionality, depending only on the parameters of the selected material. Thus, the value of the scale factor, which is equal to Δ*U_out_*/Δ*a*, quadratically increases with increasing length of the piezoelectric transducer and does not depend on its cross section. Therefore, the needle shape of the sensing element is optimal from the point of sensitivity. In this case, there are opportunities for microminiaturization of sensors.

## 3. Finite Element Modeling

To test the efficiency of the proposed design, as well as to evaluate some of its potential characteristics, a finite-element model of the piezoelectric transducer was created. Its calculation was carried out in OOFELIE::Multiphysics [6].

The model consisted of two cylindrical piezoelectric transducers 2 mm in diameter, 10 mm in length, and longitudinal polarization. The end of one of them was rigidly fixed and a harmonic voltage of 1 V and a frequency of 20 kHz was applied to it. The second end, which is common for the two transducers, was connected to null potential and the remaining end of the second converter was the surface of equal potentials and served as the source of the output signal. In addition to the electric signal, a constant linear acceleration *a* directed as it is shown in Figure 1, was applied to the model. As a material for piezoelectric transducers, we used CTS-19 with the following characteristics [7]: density of 7500 kg/m^3^; piezoelectric modules *d*_31_ = −155 × 10^−12^ C/N, *d*_33_ = 360 × 10^−12^ C/N; coefficients of electromechanical coupling *Kp* = 0.56, *K*_31_ = 0.29, *K*_33_ = 0.64. To take the charge leakage effect into account, a serial RC dipole between the output electrodes and the ground was introduced with the following parameters: capacitance *C* = 3.3 pF, resistance *R* = 10^8^ Ohm. It gives a value of leakage RC constant equal to 3.3 × 10^−4^ s, which is 330 times larger than the period of excitation signal.

**Figure 3:**
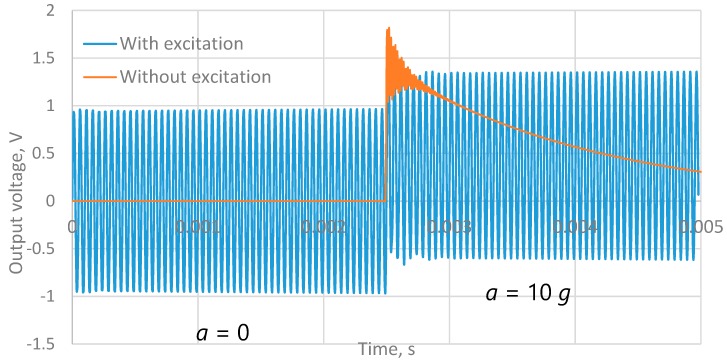
Output signal at different values of acceleration (simulation results).

The time variation of the electric potential on the surface of the free end of the piezoelectric transducer was evaluated. As expected, the midline of the harmonic signal generated by the exciter has shifted (Figure 3). At the same time, during the entire time of acceleration acting, it does not change, despite the possible leakage of charge. A line depicting a change of output signal without excitation but with other parameters the same is also plotted for comparison. Thus, the operational performance of the proposed concept is confirmed.

**Figure 4:**
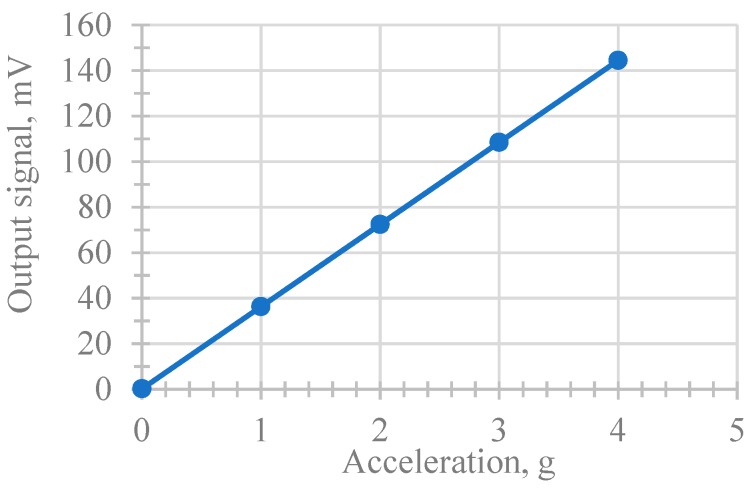
Proposed sensor sensitivity (modeling results).

Further various values of acceleration acting along the main and orthogonal axes were specified. On the basis of the obtained results, the output characteristic shown in Figure 4 was plotted. The output signal is the shift of the midline of the harmonic signal from its value at *a* = 0.

As can be seen from Figure 4, the output characteristic is linear. The value of the scale factor along the *x*-axis (measuring one) was 36 mV/g. The sensitivity along the *y* and *z* axes was 0.29 and 0.12 mV/g, respectively.

## 4. Conclusions

The proposed design of the piezoelectric accelerometer has a number of significant advantages:

- wide frequency range (from 10^−5^ Hz);
- high detection limit (up to 10^−5^ g);
- absence of moving parts;
- simplicity in manufacturing;
- low cross-sensitivity;
- possibility of miniaturization.

Due to the abovementioned benefits the sensor should find its niche in the existing accelerometer market and, therefore, its development is economically sensible.

Using the finite-element analysis, the operability of the proposed accelerometer design was confirmed and the scaling factors for all three axes (36, 0.29, and 0.12 mV/g) were evaluated. In the future, it is planned to manufacture a laboratory model of a proposed piezoelectric accelerometer and find out its operational characteristics.
